# The relationship between organizational trust and voice behavior among neonatal intensive care unit nurses in tertiary A hospitals in Sichuan Province: the mediating role of career resilience

**DOI:** 10.3389/fpubh.2024.1505641

**Published:** 2024-12-11

**Authors:** Xiujuan Zhang, Xi Huang, Yanling Hu, Qiong Chen, Xiufang Zhao

**Affiliations:** ^1^Department of Neonatology Nursing, West China Second University Hospital, Sichuan University, Chengdu, China; ^2^Key Laboratory of Birth Defects and Related Diseases of Women and Children, Sichuan University, Ministry of Education, Chengdu, China; ^3^Department of Nursing, West China Second University Hospital, Sichuan University, Chengdu, China

**Keywords:** career resilience, neonatal nurses, organizational trust, voice behavior, mediating effect

## Abstract

**Background:**

Neonatal intensive care unit (NICU) nurses face immense pressure, yet research on their voice behavior and the motivational mechanisms behind it is limited. Specifically, the impact of organizational trust and career resilience on this behavior has not been thoroughly explored.

**Aim:**

This study aims to examine the relationship between organizational trust and voice behavior in NICU nurses, with career resilience acting as a mediating factor, providing empirical evidence for nursing management.

**Methods:**

A multicenter cross-sectional survey was conducted from January to June 2023, involving 422 neonatal nurses from tertiary hospitals in Sichuan Province, China. Data were collected using a self-designed questionnaire, a voice behavior scale, an organizational trust scale, and a career resilience scale. Hierarchical regression and structural equation modeling (SEM) were employed to analyze the relationships among the variables.

**Results:**

Hierarchical regression analysis revealed that organizational trust (*β* = 0.28, *p* < 0.001) and career resilience (*β* = 0.45, *p* < 0.001) significantly predicted voice behavior. Mediation analysis using structural equation modeling confirmed that career resilience mediated the relationship between organizational trust and voice behavior, with a mediation effect of 0.340, accounting for 44.8% of the total effect. The structural model demonstrated good fit indices (CFI = 0.962, RMSEA = 0.045), indicating the robustness of the proposed model.

**Conclusion:**

Organizational trust significantly influences NICU nurses’ voice behavior, with career resilience playing a critical mediating role. Enhancing organizational trust and fostering career resilience among NICU nurses can improve their willingness to engage in voice behavior, ultimately leading to better healthcare outcomes.

**Implications for nursing management:**

Nursing managers should foster a trusting and supportive work environment to improve nurses’ job satisfaction and organizational commitment. This can be achieved by enhancing psychological empowerment and promoting positive interactions between nurses, the organization, and leadership. Such an environment helps reduce burnout and strengthens career resilience. Increased resilience enables nurses to better manage clinical pressures and challenges, elevating their career expectations and enhancing their willingness to engage in work. This, in turn, promotes innovation, active participation, and improved voice behavior, ultimately contributing to organizational success.

## 1 Introduction

The Neonatal Intensive Care Unit (NICU) primarily cares for newborns within the first 28 days of life who are at risk of life-threatening conditions or have already developed severe illnesses. Due to the rapid changes in their conditions and high mortality rates, these patients require continuous and close monitoring by medical staff, with NICU nurses playing a crucial role. The high workload, frequent emergencies, and strong reliance on teamwork result in NICU nurses experiencing significantly higher levels of stress compared to nurses in general pediatrics or adult wards, which poses a risk of stress-related occupational hazards (1–3). As the bridge between doctors and newborns, NICU nurses may experience diminished empathy under high work pressure, leading to insensitivity, burnout, and moral detachment when assessing and intervening in the life care and clinical treatment of patients. This can reduce the efficiency of nursing care and potentially degrade the quality of work (4). Such adverse effects are linked to higher in-hospital mortality rates, hospital-acquired infections, medication errors, falls, and treatment abandonment (5). Additionally, increased work pressure is associated with a higher incidence of needlestick and sharps injuries among nurses (6). Nurses under excessive stress are also more likely to experience mental health issues such as anxiety, depression, insomnia, and post-traumatic stress disorder (PTSD) (7).

In this context, effectively reducing work-related stress and improving job satisfaction among NICU nurses has become a critical issue for hospital management. Studies have shown (8–10) that encouraging nurses to actively participate in decision-making and workplace improvements, also known as voice behavior, is an effective strategy for reducing stress and enhancing job satisfaction. Voice behavior refers to the communicative actions individuals take to proactively express specific suggestions aimed at improving existing organizational issues or promoting organizational development, this behavior reflects an individual’s sense of ownership and emotional expression, serving as an effective process of decision-making and expression, which benefits both personal and organizational long-term growth (8). In the workplace, voice behavior entails speaking out about current issues, which can grant nurses, especially frontline non-management nurses, a greater sense of involvement and decision-making power. This helps to enhance job satisfaction and reduce work stress (9). In practice, hospitals that encourage nurses to provide suggestions and participate in decision-making report significantly higher job satisfaction and psychological health levels among their nurses compared to those that do not encourage such participation. Nurses in these supportive environments also report significantly lower work stress (10). Involvement in nursing management helps nurses better understand and implement work processes and requirements, reducing resistance and uncertainty during the adoption of management decisions. Studies have shown (11) that regularly holding meetings for nurses to discuss work improvements and promote voice behavior not only enhances the quality of care but also significantly reduces nurse turnover rates and levels of professional burnout.

However, voice behavior does not occur spontaneously, it is influenced by various factors. Among them, organizational trust and career resilience are two key variables. Organizational trust refers to employees’ subjective perception of their organizational atmosphere, specifically their evaluation of whether the work environment is safe or friendly. According to research (12, 13), organizational trust can effectively promote cooperation and communication in the workplace, reduce friction, and enhance job satisfaction, thereby having a positive impact on voice behavior. For NICU nurses, organizational trust is their subjective assessment of the hospital or department atmosphere, primarily measured by their self-evaluation of the safety and friendliness of the hospital or department environment. Studies have found (14–18) that organizational trust can act as a psychological mechanism positively influencing voice behavior, suggesting that increasing organizational trust can lead to more voice behavior. Nurses’ career resilience refers to their ability to positively self-regulate and recover from trauma when facing threats and pressures in their professional careers (19). Enhancing career resilience can promote proactive changes among employees, driving innovation and beneficial development within the organization (20). Additionally, literature reports that voice behavior may have a reciprocal positive effect on career resilience. When nurses’ proactive suggestions receive favorable feedback, it can boost their confidence and self-efficacy, thereby increasing job satisfaction and further enhancing career resilience (21).

Although existing research has shown that organizational trust and career resilience positively influence voice behavior (22, 23), the intrinsic motivations and mechanisms of voice behavior, especially among NICU nurses in high-pressure environments, remain underexplored. Most current studies focus on the direct relationship between organizational trust and voice behavior, neglecting other factors that may play a mediating role. Career resilience, as an important psychological regulation mechanism, may play a crucial mediating role between organizational trust and voice behavior. Therefore, this study aims to fill this gap by exploring the relationships among organizational trust, career resilience, and voice behavior in the NICU work environment, and by developing a corresponding theoretical framework. The theoretical framework for this study is based on the following theoretical models:

### 1.1 Social exchange theory

Based on Social Exchange Theory (24, 25), employee behavior is closely tied to the level of trust they have in their organization. This theory posits that when individuals perceive strong support from their organization or colleagues, they feel a sense of obligation to reciprocate by engaging in positive behaviors, such as voice behavior or enhanced job performance. In the context of NICU nurses, organizational trust can foster a sense of responsibility and loyalty, which, in turn, encourages them to actively participate in voice behavior. Given the high-stress nature of NICU work, where nurses must navigate complex challenges and work under constant pressure, it is reasonable to expect that organizational trust may have a particularly strong influence on their willingness to engage in voice behavior. Therefore, we hypothesize that: Hypothesis 1 (H1): There is a positive correlation between organizational trust and voice behavior among NICU nurses.

### 1.2 Career resilience theory

Career resilience, as a psychological adjustment mechanism, helps employees maintain a positive mindset and fosters self-regulation and organizational innovation when facing work-related stress and challenges (20, 26). Research shows that person-job match in areas such as control, community, and value plays a crucial role in enhancing employees’ job embeddedness and performance, with career resilience serving as a key moderating factor in the relationship between employees and their organization (26). In this context, when nurses experience organizational trust and support, their career resilience is likely to increase, enabling them to better cope with challenges and exhibit more positive behaviors (27). In high-stress environments such as NICUs, career resilience is especially important because it enables nurses to maintain mental well-being and adapt to constant changes and pressures. Based on this, we propose Hypothesis 2 (H2): There is a positive correlation between organizational trust and career resilience among NICU nurses. Social exchange theory emphasizes that when employees perceive support and trust from their organization, they feel an obligation to reciprocate by exhibiting positive behaviors. Career resilience theory, on the other hand, provides a psychological adjustment mechanism that helps employees cope with stress and challenges while maintaining a positive mindset. Based on this, we propose that organizational trust enhances nurses’ career resilience, enabling them to better cope with work-related stress and challenges, thereby increasing their willingness to engage in voice behavior. Therefore, we propose Hypothesis 3 (H3): Career resilience is positively correlated with voice behavior among NICU nurses.

Mediation Model: Based on the above theories, we believe that career resilience may play a mediating role between organizational trust and voice behavior. Organizational trust can enhance nurses’ career resilience, which in turn further promotes voice behavior. This is particularly relevant in the NICU context, where the high-pressure environment demands both emotional resilience and proactive engagement in workplace communication. Drawing on Social Exchange Theory and the concept of felt obligation, when nurses perceive support and trust from the organization, their career resilience and job embeddedness are strengthened, giving them greater motivation to express their ideas and engage in voice behavior (25–27). Additionally, research shows that a higher person-job match in areas such as control, community, and value increases both career resilience and job embeddedness, further encouraging positive behaviors (26). Based on this, we propose Hypothesis 4 (H4): Career resilience mediates the relationship between organizational trust and voice behavior among NICU nurses.

**Figure 1:**
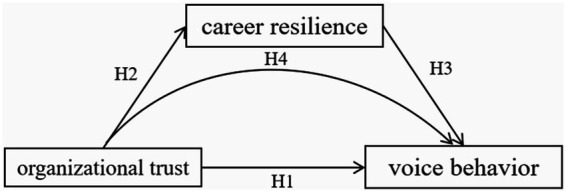
Hypothesized model.

The hypothesized model for this study is presented in Figure 1.

Through an empirical analysis of NICU nurses from tertiary hospitals in Sichuan Province, this study aims to test the above hypotheses and explore the specific mechanisms between the related variables. This will not only deepen the understanding of the psychological stress and work behaviors of NICU nurses, but also provide hospital management with new strategic insights to improve nurse job satisfaction, reduce work-related stress, and ultimately enhance the quality of healthcare services.

## 2 Methods

### 2.1 Participants

This study used stratified cluster sampling to select NICU nurses working in tertiary public hospitals in Sichuan Province from January to September 2023. Inclusion criteria were: licensed and actively working nurses; no physical or psychological conditions affecting clinical work during the survey period; no major life changes (e.g., divorce, death of a relative, severe illness, serious injury, or significant property loss) in the past six months; and voluntary participation in the study. Exclusion criteria were: nurses currently undergoing standardized training or rotating through departments, trainee nurses, probationary nurses, retired or rehired retired nurses, and those who have previously participated in similar surveys. Additionally, questionnaires completed in less than five minutes were excluded.

This study is a multicenter cross-sectional study. The sample size was determined based on 5 to 15 times the number of questionnaire items, plus an additional 10 to 20% to account for non-response rates (28). This study includes three scales: the Voice Behavior Scale (10 items), the Organizational Trust Scale (13 items), and the Career Resilience Scale (32 items), with the largest scale containing 32 items. To ensure sufficient representativeness and robustness, the sample size was initially estimated using the rule of thumb that the sample size should be 10 times the number of items in the largest scale, resulting in a minimum required sample size of 320 participants. Considering a 20% non-response rate, the final minimum sample size was determined to be 384 participants. To further ensure that the sample size had sufficient statistical power to detect the effects, a power analysis was conducted. Based on Cohen’s (29) guidelines, with a significance level of *α* = 0.05 and a medium effect size (0.3), the common statistical power of 0.80 (Power = 0.80) was selected. Using statistical tools such as G*Power, the calculated minimum sample size requirement was approximately 380 participants.

### 2.2 Measurement tools

#### 2.2.1 General information questionnaire

Designed by the researchers, this questionnaire includes items on gender, age, ethnicity, only-child status, personal health condition, marital status, number of children, location of the workplace, nature of the workplace, educational background, professional title, position, years of experience in the neonatal department, and any significant family or personal events in the past six months.

#### 2.2.2 Voice behavior scale

Developed by Liang et al. (30) within the context of Chinese enterprises, this scale is widely used among nurses and public sector employees in China. It is currently the most frequently used and localized research tool in the field of organizational behavior studies, with a Cronbach’s *α* coefficient ranging from 0.84 to 0.90. The questionnaire consists of 10 items, divided into two dimensions: promotive voice behavior (5 items) and prohibitive voice behavior (5 items). It uses a 5-point Likert scale, where 1 indicates “never” and 5 indicates “always,” with a total possible score of 50. Higher scores indicate a higher frequency of voice behavior. In this study, the overall Cronbach’s *α* coefficient for the questionnaire was 0.951, with Cronbach’s α values of 0.950 for promotive voice behavior and 0.911 for prohibitive voice behavior.

#### 2.2.3 Organizational trust scale

Developed by Chen Jinggang (31), this scale is widely used among nurses and state-owned enterprise employees, demonstrating good reliability and validity with a Cronbach’s *α* coefficient of 0.936. The scale comprises 13 items across three dimensions: trust in the organization (5 items), trust in leadership (4 items), and trust in colleagues (4 items). It uses the same 5-point Likert scale as the Voice Behavior Scale, with a total score of 65 points. Higher scores indicate better organizational trust. In this study, the overall Cronbach’s *α* coefficient for the questionnaire was 0.968, with Cronbach’s *α* values of 0.942 for organizational trust, 0.962 for leadership trust, and 0.941 for colleague trust.

#### 2.2.4 Career resilience scale

Developed specifically for nursing staff by Xuemei et al. (32), this questionnaire has an overall Cronbach’s *α* coefficient of 0.956, with dimension-specific Cronbach’s α coefficients ranging from 0.790 to 0.888, and a test–retest reliability of 0.86. The scale comprises 32 items across six dimensions: career emotion (4 items), teamwork (7 items), adaptability (6 items), self-efficacy (5 items), career goals (4 items), and learning and growth (6 items). It uses a 5-point Likert scale, where 1 indicates “completely disagree” and 5 indicates “completely agree,” with higher scores indicating higher levels of career resilience. In this study, the overall Cronbach’s *α* coefficient for the questionnaire was 0.988, with dimension-specific Cronbach’s α coefficients ranging from 0.932 to 0.964.

### 2.3 Survey methodology and quality control

This study used hospitals as sampling units and adopted a stratified cluster sampling method. Among the 2,534 medical institutions registered with the Sichuan Provincial Health Commission, 120 are Class IIIA non-profit, fully state-owned institutions under the administration of health authorities. Departmental information was reviewed on the official websites of these 120 institutions, revealing 77 facilities that treat newborns, with 54 having dedicated neonatal departments. Two hospitals were excluded based on verification from the Sichuan Neonatal Collaboration Platform, as they no longer maintain standalone neonatal departments despite outdated information on their websites, leaving 52 eligible target institutions. Nurse counts for the neonatal departments were gathered from hospital websites and the Sichuan Neonatal Collaboration Platform; where nurse numbers were not explicitly stated, estimates were made using a nurse-to-patient ratio of 1:1.5 in Level III neonatal wards. Data stratification was conducted according to the geographic area and population proportion of the sampled regions to ensure regional representativeness.

The target population from hospitals meeting the inclusion and exclusion criteria was surveyed using an electronic questionnaire via the Jinshuju platform (website: https://jinshuju.net). The study received approval from the Hospital Medical Ethics Committee [Medical Research 2023 Ethics Approval No. (042)], and written authorization was obtained from Professor Yang Zhaoxia, the developer of the Career Resilience Scale. Before the survey began, researchers explained the purpose, content, and completion requirements of the questionnaire to the participants, who then signed electronic informed consent forms. The questionnaires were filled out anonymously to ensure data confidentiality.

The questionnaire included standardized instructions, with all items required to be completed. A total of 575 questionnaires were collected, achieving a 100% response rate. After downloading the data, researchers checked the completeness and validity of the questionnaires, excluding those that did not meet the criteria: 4 questionnaires from probationary nurses, 1 from a retired nurse (aged 60), 20 from non-tertiary public hospitals, 21 from nurses with physical or psychological conditions potentially affecting clinical work, 58 from individuals experiencing major personal or family events in the past 6 months, and 49 completed in less than 5 min. Ultimately, 422 valid questionnaires were obtained, with an effective rate of 73.39%.

### 2.4 Statistical analysis

Descriptive statistics and correlation analysis were performed using SPSS 26.0. Measurement data conforming to a normal distribution were presented as ( $\bar{x}\pm s$ ), and count data were expressed as proportions. Inter-group comparisons were conducted using independent *t*-tests and analysis of variance (ANOVA). Reliability testing was performed to assess internal consistency. For structural validity, exploratory factor analysis was initially conducted, followed by confirmatory factor analysis based on the results. Pearson correlation coefficient analysis was used for correlation analysis. AMOS 26.0 software was utilized to establish the mediation effect model, and model fitting evaluation and modifications were performed. A *p*-value of less than 0.05 was considered statistically significant.

## 3 Results

### 3.1 Basic information of survey participants

Among the 422 nurse respondents, 419 were female (99.3%) and 3 were male (0.7%). The age distribution was as follows: 135 nurses (32.0%) were under 30 years old, 223 nurses (52.8%) were between 30 and 39 years old, 58 nurses (13.8%) were between 40 and 49 years old, and 6 nurses (1.4%) were 50 years old or above. Ethnic distribution included 405 Han (96.0%), 13 Tibetan (3.1%), and 4 Yi (0.9%) nurses. Regarding family background, 132 nurses (31.3%) were only children, while 290 nurses (68.7%) had at least one sibling. Marital status was as follows: 319 nurses (75.6%) were married, 92 nurses (21.8%) were single, and 11 nurses (2.6%) were divorced. In terms of children, 132 nurses (31.3%) had no children, 197 nurses (46.7%) had one child, and 93 nurses (22.0%) had two children. Educational qualifications were as follows: 346 nurses (82.0%) had a bachelor’s degree, 11 nurses (2.6%) had a master’s degree or higher, and 65 nurses (15.4%) had an associate degree. The workplaces of the participants were distributed as follows: 321 nurses (76.1%) worked in general hospitals, and 101 nurses (23.9%) worked in specialized hospitals. Professional titles included 193 nurses (45.7%) with the title of Nurse Practitioner, 175 nurses (41.5%) with the title of Senior Nurse Practitioner, and 22 nurses (5.2%) with senior professional titles. The majority of the respondents were frontline nurses, comprising 79.4% (335/422) of the total. The work experience distribution was concentrated in the 0–5 years and 6–10 years categories, representing 36.7% (155/422) and 30.6% (129/422) of the respondents, respectively.

### 3.2 Questionnaire reliability and validity testing

Reliability analysis was conducted separately for the voice behavior scale, organizational trust scale, and career resilience scale. The results showed that the overall Cronbach’s *α* for the voice behavior scale was 0.951, with Cronbach’s *α* values of 0.950 and 0.911 for promotive and prohibitive voice, respectively. For the organizational trust scale, the overall Cronbach’s *α* was 0.968, with *α* values of 0.942, 0.962, and 0.941 for the dimensions of organizational trust, leader trust, and colleague trust, respectively. The overall Cronbach’s *α* for the career resilience scale was 0.988, with each dimension’s Cronbach’s *α* exceeding 0.932; the item-level Cronbach’s α values were around 0.987, with no significant drop when any item was removed, indicating good reliability for all three scales.

In the validity analysis, the KMO and Bartlett’s sphericity tests were conducted for the voice behavior, organizational trust, and career resilience scales. The KMO values were 0.938, 0.947, and 0.975, respectively, indicating a high degree of commonality among variables in each questionnaire. Bartlett’s sphericity test results were all significant at *p* < 0.001, suggesting significant differences in the correlation matrices, allowing further factor analysis. Confirmatory factor analysis results indicated that the model fit indices for the voice behavior scale were CMIN/DF = 3.352, RMSEA = 0.075, TLI = 0.974, CFI = 0.985, NFI = 0.979, and SRMR = 0.000. For the organizational trust scale, the indices were CMIN/DF = 4.661, RMSEA = 0.093, TLI = 0.958, CFI = 0.973, NFI = 0.966, and SRMR = 0.000. For the career resilience scale, the indices were CMIN/DF = 3.320, RMSEA = 0.074, TLI = 0.939, CFI = 0.948, NFI = 0.928, and SRMR = 0.000. These indices indicate a good model fit for each scale, demonstrating strong structural validity across all three scales.

### 3.3 Common method bias test

Since the data in this study were collected through self-reports from nurses, there is a potential risk of common method bias (CMB), which may result in systematic measurement errors due to the single-source nature of the data. To address this issue, this study employed the common method variance detection technique known as the single-factor confirmatory factor analysis (CFA), one of the commonly used methods for detecting CMB. This technique examines the model’s goodness-of-fit by assigning all measured items to one latent factor. If all items can be explained by a single factor and the model fits well, this indicates the presence of substantial CMB; conversely, if the single-factor model fits poorly, it suggests that the items are not from the same factor and the CMB is minimal (33).

In this study, a theoretically expected multi-factor model (M1) was first constructed, and its fit indices were calculated as the baseline. The fit indices for the M1 model were as follows: CMIN/DF = 4.146, RMSEA = 0.087, TLI = 0.967, CFI = 0.980, and SRMR = 0.000, indicating a good fit and showing that the data can be explained by the expected multi-factor structure. Subsequently, a single-factor model (M2) was constructed by forcing all items into one factor, and its fit indices were calculated. The fit indices for the M2 model were as follows: CMIN/DF = 8.573, RMSEA = 0.134, TLI = 0.641, CFI = 0.654, and SRMR = 0.060, indicating a poor fit. In particular, the key fit indices such as RMSEA and CFI were far below acceptable standards, suggesting that the items cannot be explained by a single factor.

By comparing the fit indices of the two models, it is evident that the fit of M1 is significantly better than that of M2, indicating that the measurement items in this study are not solely derived from a single factor and that there is no significant common method bias in the data.

### 3.4 Scores of voice behavior, organizational trust, and career resilience among NICU nurses with different demographic characteristics

The results of this study show that there are statistically significant differences in voice behavior among NICU nurses based on age, marital status, and number of children (*p* < 0.05). Specifically: Voice behavior increases with age up to 50 years. Unmarried nurses exhibit less voice behavior compared to married or divorced nurses. Nurses with more children tend to display more voice behavior. Additionally, there are significant differences in voice behavior based on professional title, position, and years of experience in the neonatal unit (*p* < 0.05): Nurses in higher positions exhibit more voice behavior. Nurses with more than 15 years of experience demonstrate higher voice behavior compared to those with 6–15 years or 0–5 years of experience. Nurses with higher professional titles show an increase in voice behavior as their titles advance. The differences in promotive and prohibitive voice behavior are only observed among those with senior titles, with senior nurses exhibiting significantly higher levels of prohibitive voice behavior compared to other titles.

The study results indicate that there are no statistically significant differences in organizational trust among participants with different demographic characteristics (*p* > 0.05). However, nurses holding positions as head nurses or deputy head nurses exhibit significantly higher organizational trust compared to those in other positions (*p* < 0.05). There is no significant difference in organizational trust between key staff/team leaders and frontline nurses.

Differences in career resilience are only evident in relation to the number of children. Nurses with children, regardless of the number, exhibit higher career resilience compared to those without children, and this difference is statistically significant (*p* < 0.05).

### 3.5 Correlation analysis of voice behavior, organizational trust, and career resilience among nurses

Pearson correlation analysis results show significant positive correlations between total voice behavior scores and total organizational trust scores (*r* = 0.654, *p* < 0.01), as well as between total voice behavior scores and total career resilience scores (*r* = 0.693, *p* < 0.01). Additionally, there is a significant positive correlation between total organizational trust scores and total career resilience scores (*r* = 0.741, *p* < 0.01). Furthermore, both organizational trust and career resilience are positively correlated with promotive voice behavior and prohibitive voice behavior (see Table 1).

**Table 1:** Correlation analysis of voice behavior with organizational trust and career resilience (*n* = 422).

Variable	M ± SD	A	A1	A2	C	F
A Total voice behavior	41.52 ± 7.11	1				
A1 Promotive voice behavior	21.26 ± 3.72	0.94**	1			
A2 Prohibitive voice behavior	20.26 ± 3.83	0.94**	0.77**	1		
C Organizational trust total score	55.75 ± 9.08	0.65**	0.62**	0.62**	1	
F Career resilience total score	135.07 ± 20.68	0.69**	0.66**	0.64**	0.74**	1

**P < 0.01.

### 3.6 Multivariate hierarchical regression analysis

A hierarchical regression analysis was conducted with demographic variables as control variables, and organizational trust and career resilience as independent variables, to predict total voice behavior, promotive voice behavior, and prohibitive voice behavior. The results show that among demographic variables, the number of children can positively predict voice behavior, while job position can negatively predict voice behavior. Both organizational trust and career resilience can significantly positively predict voice behavior, explaining 45.7% of the variance (see Table 2).

**Table 2:** Hierarchical regression analysis of organizational trust and career resilience on total voice behavior (*n* = 422).

Predictor variables	Model 1		Model 2		Model 3	
	*β*	*t*	*β*	*t*	*β*	*t*
Age	0.13	1.73	0.13	2.32*	0.15	3.04**
Marital status	−0.10	−1.60	−0.10	−2.23*	−0.08	−2.00*
Number of children	0.17	2.71**	0.13	2.81**	0.07	1.72
Professional title	−0.10	−1.42	−0.08	−1.62	−0.07	−1.53
Job position	−0.12	−2.08*	−0.07	−1.62	−0.09	−2.32*
Years of work experience	0.04	0.61	0.03	0.57	0.01	0.28
Organizational trust total score			0.61	18.18***	0.28	6.28***
Career resilience					0.45	9.87***
*R^2^*	0.17		0.54		0.63	
Adjusted *R^2^*	0.16		0.53		0.62	
*∆R^2^*	0.17		0.37		0.09	
*F*	12.10***		60.34***		76.99***	

***P < 0.001; **P < 0.01; *P < 0.05.

### 3.7 Mediation effect analysis of career resilience between organizational trust and voice behavior

**Figure 2:**
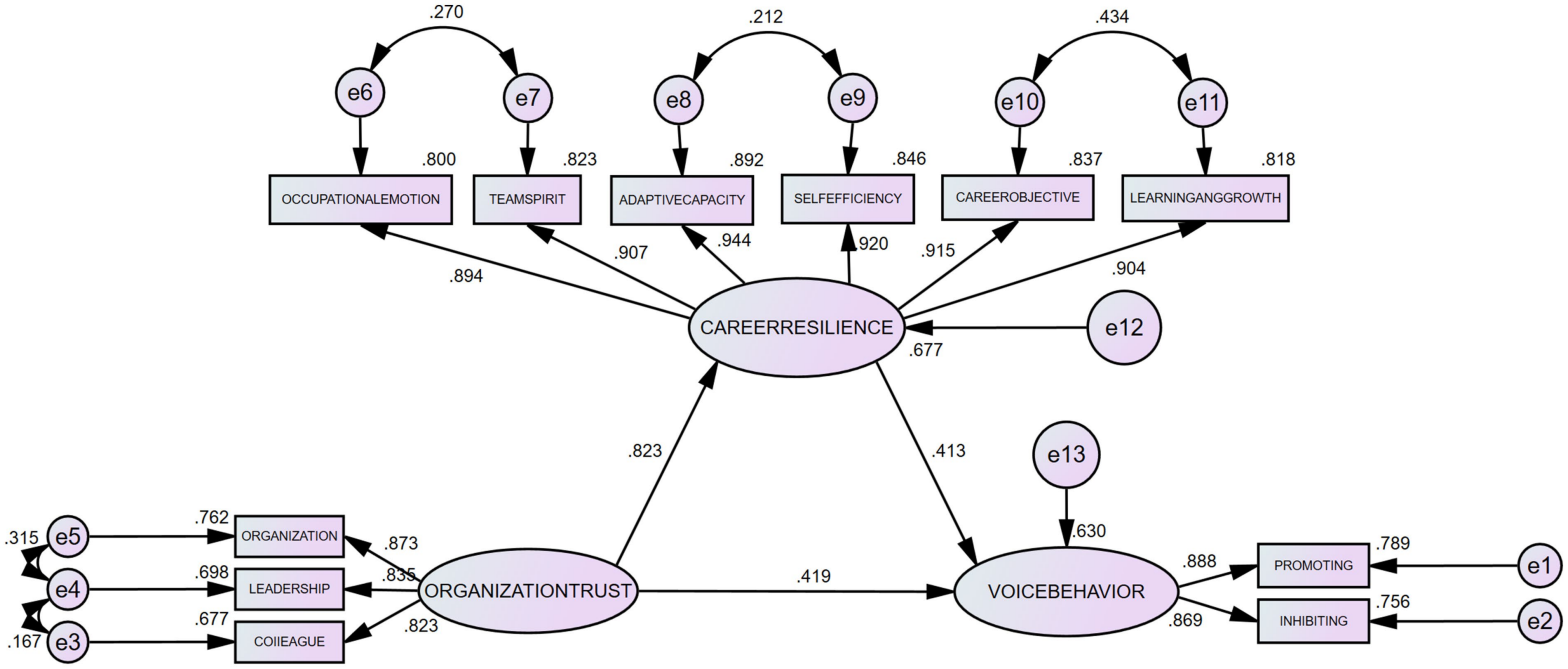
Mediation effect of career resilience between organizational trust and voice behavior.

Using AMOS 26.0 software, the mediation effect between the questionnaires was analyzed, controlling for demographic variables. The effect relationships and model fit indices among the three questionnaires (Voice Behavior Scale, Organizational Trust, Scale, and Career Resilience Scale) were as follows: CMIN/DF (Chi-square to degrees of freedom ratio) = 3.990, RMSEA (Root Mean Square Error of Approximation) = 0.084, TLI (Tucker-Lewis Index) = 0.969, CFI (Comparative Fit Index) = 0.980, and SRMR (Standardized Root Mean Square Residual) < 0.001. The overall model fit was satisfactory (see Figure 2).

The analysis results show that the path coefficient from organizational trust to career resilience is 0.823, with *p* < 0.001, indicating a significant positive impact of organizational trust on career resilience, thereby confirming Hypothesis H2. Additionally, the path coefficients from organizational trust and career resilience to total voice behavior are 0.419 and 0.413, respectively, both with *p* < 0.001, indicating that both organizational trust and career resilience had a significant positive impacts on voice behavior. Thus, Hypotheses H1 and H3 were also confirmed.

“To ensure the accuracy and robustness of the results, this study employed 5,000 samples in the Bootstrap test (34). The findings reveal that career resilience plays a significant mediating role between organizational trust and voice behavior.” Specifically, the standardized mediating effect of career resilience was 0.340 [95% CI (0.241, 0.524), *p* = 0.001], indicating a significant mediation effect. Furthermore, the mediation effect of career resilience accounted for 44.8% of the total effect of organizational trust on voice behavior, highlighting the crucial role that career resilience plays in promoting voice behavior. This finding indicates that when enhancing organizational trust, the pathway of indirectly fostering voice behavior through increased career resilience is particularly important. The fact that career resilience accounts for nearly half of the mediation effect suggests that implementing trust-enhancing measures in organizations—such as fair and transparent management practices and fostering a culture of positive feedback—can significantly improve employees’ adaptability and stress management capabilities, which in turn encourages them to offer constructive suggestions to the organization. This path analysis not only validates the theoretical model but also provides concrete intervention points for management practice.

## 4 Discussion

### 4.1 Current status of voice behavior, organizational trust, and career resilience among NICU nurses

Scholars generally believe that voice behavior tends to increase with age. As nurses gain more work experience, older nurses develop stronger critical thinking skills and are better able to identify problems in their work. Additionally, with age, nurses become more adept at weighing the pros and cons and choosing the right time to express their opinions without fear of speaking up or the potential risks involved. This view is supported by several studies, such as the research by Kang Huihui (35), which found that public servants over the age of 50, due to greater emotional stability and reduced risk considerations, are more likely to engage in voice behavior, scoring the highest in this area. The study found that there was no significant difference in voice behavior between NICU nurses aged 50 and above and those below 50 (43.33 ± 8.19 vs. 46.14 ± 5.28). This result contrasts with previous research, which may be related to the specific nature of NICU nursing work. Older nurses typically engage less in frontline clinical tasks, which may lead to a decrease in their ability to assess and innovate, resulting in fewer opportunities and motivations to engage in voice behavior. For the pre-retirement group, studies have shown that (36) their job satisfaction and perceived value are lower, which may impact their willingness to engage in voice behavior. Therefore, when implementing strategies to promote voice behavior, factors such as work content, environment, and psychological state, in addition to age, should be considered comprehensively. In contrast, marital status showed a significant impact on voice behavior in this study. Unmarried nurses had the lowest voice behavior scores, while divorced nurses scored higher in terms of suppressive voice behavior compared to married nurses. This finding is consistent with Xiang’s (37) research and may be related to the psychological state and family burdens of the nursing staff. Divorced nurses, perhaps due to having fewer family obligations, may experience a more relaxed mindset, allowing them to invest more energy in their work and approach workplace issues more proactively.

In terms of job title, there was no significant difference in voice behavior between nurses and junior nurses. However, as the job title increased, there was a significant rise in voice behavior, especially among those with senior titles. This may reflect the growing ability of nurses to solve problems and propose improvements as they accumulate more work experience.

This study found that demographic variables (such as age, gender, years of service, and educational level) had no significant impact on organizational trust among NICU nurses in tertiary hospitals in Sichuan Province. This may be closely related to the unique work environment in NICUs, where nurses tend to rely more on team spirit and professional competence to build trust, rather than individual demographic characteristics. In contrast, job position significantly influences organizational trust, especially among nurses in managerial positions (e.g., head nurses or deputy head nurses), where organizational trust is more prominent. This may be because nurses in managerial roles are more involved in departmental decision-making and shaping the department’s culture, which strengthens their trust in the organization. Organizational trust is a subjective experience of the atmosphere within the organization, influenced by various factors, including individual personality, work atmosphere, leadership style, compensation and performance, and staffing levels (38). To enhance organizational trust among non-managerial nurses, hospital management should focus on building trust across different job levels. The following strategies can be considered:

1. Enhance communication and transparency: Research highlights that creating opportunities for nurses to voice their concerns and opinions fosters a sense of inclusion and trust (26). Hospital leadership should implement regular meetings and feedback channels, ensuring that nurses at all levels feel valued and have platforms to share their perspectives (27).
2. Provide equal training and development opportunities: Studies emphasize the significance of equitable career development opportunities in building trust (26). Hospital management should ensure that all nurses, regardless of their position, have access to training and professional growth opportunities, which can increase trust in management and hospital policies (25).
3. Establish a fair promotion system: Fairness in promotion systems has been shown to strongly influence job embeddedness and organizational commitment (26). A transparent and fair promotion system should be developed, where nurses clearly understand the rewards for their efforts and the pathways for career advancement. This, in turn, can significantly enhance their trust in the organization (27).

By implementing these strategies, hospital leadership can expect an overall increase in organizational trust, not only within the NICU but also across other departments. This enhanced trust can lead to improved team collaboration, greater job embeddedness, and increased operational efficiency (26, 27).

The study also found that nurses with children exhibited higher career resilience compared to those without children. This may be related to the greater family economic pressure and more experience in handling stressors faced by nurses with children, which enhances their ability to cope with work-related stress. Additionally, nurses with children often possess stronger self-management skills and psychological resilience, enabling them to better navigate complex work environments and demonstrate higher career resilience. Career resilience has a significant positive impact on nurses’ job satisfaction, job burnout, and turnover rates (19). Therefore, to enhance nurses’ career resilience, managers should pay particular attention to those without children and implement supportive measures, such as providing psychological counseling and stress management training, to help them better cope with career challenges and improve their career resilience.

### 4.2 The relationship between organizational trust, career resilience, and voice behavior

This study shows a significant positive correlation between organizational trust and voice behavior among NICU nurses, with organizational trust positively predicting voice behavior.

1. Organizational Trust: Establishing a fair and just organizational culture is crucial for promoting voice behavior. Research by van Marum et al. (39) shows that a correct, fair, and transparent reward and punishment system helps enhance organizational trust and promote voice behavior. In an environment filled with trust, nurses are more willing to share their ideas and opinions, believing that these suggestions may be adopted.
2. Leadership Trust: Increasing leaders’ trust in nurses helps them understand the purpose and significance of nurses’ voice behavior, thereby enhancing nurses’ self-efficacy and motivation. Studies have shown (40–42) that a supportive leadership style contributes to improving nurses’ work well-being, strengthening their trust in both leadership and the organization, and is negatively correlated with burnout.
3. Peer Trust: In the closed management model of the NICU, close cooperation and trust among colleagues help promote voice behavior. An open communication environment allows nurses to more actively offer suggestions when facing difficulties, thereby improving overall healthcare quality.

Research shows that the higher the career resilience, the more frequent the voice behavior. This aligns with the findings of Guoxue (43), who found a significant positive correlation between career resilience and positive emotions. When nurses face clinical work pressure, high career resilience helps them avoid negative coping mechanisms. Instead, they can find positive aspects in the pressure or difficulties, and view the consequences of adverse stimuli positively. This ability transforms problems into a driving force for change, reducing the fear of the consequences of voice behavior, and increasing the desire to express and suggest improvements, ultimately promoting voice behavior among nurses. There is a significant positive correlation between the total scores of organizational trust and career resilience (*r* = 0.741, *p* < 0.01), indicating that stronger organizational trust among NICU nurses is likely to result in higher career resilience. Structural equation modeling results also show that organizational trust has a significant positive impact on career resilience, consistent with the findings of many scholars (44, 45).

### 4.3 The mediating effect of career resilience on the relationship between organizational trust and nurses’ voice behavior

The mediation effect analysis of the structural equation model revealed a significant indirect effect of organizational trust on nurses’ voice behavior through career resilience, with the mediation effect of career resilience accounting for 44.8%. This finding emphasizes that organizational trust not only directly promotes voice behavior but also indirectly influences it by enhancing career resilience. Specifically, improving organizational trust can significantly boost nurses’ psychological empowerment, which is achieved through fostering positive work attitudes, such as organizational commitment and job satisfaction (46, 47). These positive attitudes help nurses maintain a proactive mindset when facing work-related difficulties and challenges, enabling them to manage stress effectively and promote personal growth and career resilience development. Moreover, the enhancement of career resilience not only strengthens nurses’ commitment to improving the work environment and service quality but also encourages them to proactively offer constructive suggestions in their daily work (39). This behavioral shift reflects an improvement in individual capability and also highlights the strengthening of team cooperation. When nurses feel supported and trusted by the organization, they are more likely to leverage the power of teamwork to collectively face and overcome work challenges, which not only reduces burnout but also enhances the overall career resilience of the team (48).

From a management perspective, these findings provide important strategic directions for hospitals and other healthcare institutions. First, by enhancing organizational trust, healthcare institutions can more effectively encourage nurses and other medical professionals to engage in voice behavior, which is essential for continuously improving the quality of care and ensuring patient safety. Second, by supporting and fostering career resilience, organizations can not only increase individual employees’ adaptability and long-term job satisfaction but also reduce staff turnover and enhance team collaboration, ultimately improving operational efficiency. Therefore, hospital administrators should consider implementing measures such as regular mental health workshops, stress management training, and offering more opportunities for staff to participate in decision-making. These are all effective ways to increase organizational trust and career resilience. Such initiatives not only promote nurses’ professional development but are also critical steps in transforming healthcare institutions into more transparent, supportive, and efficient work environments.

In conclusion, this study found that organizational trust has a significant positive impact on voice behavior among NICU nurses in Sichuan Province, with career resilience playing an important mediating role in this process. Organizational trust enhances nurses’ voice behavior by increasing their career resilience. These findings highlight the crucial role of managers in creating a supportive and trusting work environment, which not only improves nurses’ job satisfaction and organizational commitment but also promotes psychological empowerment, positive peer interactions, reduces burnout, and strengthens career resilience.

## 5 Conclusion

This study not only confirmed the positive correlation between organizational trust and voice behavior but also revealed, for the first time, the critical role of career resilience as a mediating variable among NICU nurses. This finding provides a new theoretical perspective and practical approach for enhancing nurses’ voice behavior in high-pressure healthcare environments.

## 6 Implication for nursing management

Based on these findings, hospital management can consider implementing the following specific actions:

1. Establish a Career Resilience Enhancement Program: ① Conduct Psychological Resilience Workshops: Hospitals can regularly organize training sessions for nurses, covering topics such as stress management, emotional regulation, conflict resolution, and effective communication skills to help nurses better cope with clinical pressures and work challenges. ② Provide Career Development Support: Through career counseling and development planning, assist nurses in setting and achieving their professional goals, thereby enhancing their career expectations and job satisfaction.
2. Strengthen Organizational Trust and Support: ① Increase Decision-Making Transparency and Improve Communication: Management should ensure transparency in decision-making processes and broaden channels for nurses to participate in policy-making, ensuring that their suggestions and feedback are valued and considered. ② Establish a Fair Feedback and Recognition System: Regularly provide positive feedback on nurses’ voice behavior and recognize those who contribute significantly to improving the department’s work environment and service quality.
3. Optimize the Work Environment and Welfare System: ① Improve Working Conditions: Adjust work schedules to ensure nurses have adequate rest and vacation time, and provide better workplace facilities to reduce work-related stress. ② Offer Comprehensive Support Resources: Provide mental health services, physical health support, and burnout prevention strategies to help nurses maintain their overall well-being.

By implementing these measures, hospitals can not only enhance nurses’ career resilience and voice behavior but also create a more optimized and efficient working environment. These actions will directly improve nursing quality and patient safety, guiding the hospital toward more efficient and humane healthcare services. Evidence-based management strategies will help hospitals address the various challenges of modern healthcare, while maintaining competitiveness and fostering innovation.

## 7 Limitations and future research directions

This study has several limitations. First, due to its cross-sectional design, the study is unable to establish causal relationships between organizational trust, career resilience, and voice behavior. Therefore, future research should consider adopting a longitudinal design to further verify the causal relationships and dynamic changes between these variables. Second, the data in this study were primarily based on self-reports, which may be influenced by recall bias and social desirability bias, potentially causing discrepancies between the nurses’ reported feelings and their true emotions. To minimize these biases, future studies could incorporate anonymous feedback or use multisource data, such as evaluations from supervisors or colleagues, to gain a more objective and comprehensive perspective. Finally, although hierarchical regression analysis was used in this study to control for some confounding variables, the model still explains only 62.7% of the variance, suggesting that other potential influencing factors have not been fully considered. For example, nurses’ personality traits, work stress, and nurse–patient ratios may significantly affect the results in certain contexts. Future research could incorporate variables such as nurses’ personality traits, nurse–patient ratios, hospital admission rates, and the case mix index (CMI) to further control for confounding factors and improve the explanatory power of the model. Additionally, expanding the sample size and comparing data across different regions, departments, and work environments—especially in high-pressure nursing settings such as PICUs (pediatric intensive care units), adult ICUs, and emergency departments—would help enhance the external validity and generalizability of the findings, thereby increasing their applicability across various nursing contexts.

## Data Availability

The raw data supporting the conclusions of this article will be made available by the authors, without undue reservation.
